# Intratracheal microspray delivery of iohexol for sustained lung-parenchymal enhancement in preclinical micro-computed tomography: A feasibility and acute-safety study in mice

**DOI:** 10.1371/journal.pone.0361103

**Published:** 2026-10-23

**Authors:** Sheng-Chieh Lin, Kuo-Kai Lin, Kevin Aristyo, Hong-Min Lin, Wei-Hung Liu, Erh-Hsuan Hsieh, Ching-Li Tseng

**Affiliations:** 1 Department of Pediatrics, School of Medicine, College of Medicine, Taipei Medical University, Taipei, Taiwan; 2 Division of Allergy, Asthma, and Immunology, Department of Pediatrics, Shuang Ho Hospital, Taipei Medical University, New Taipei City, Taiwan; 3 TMU Research Center of Thoracic Medicine, Taipei Medical University, Taipei, Taiwan; 4 Graduate Institute of Biomedical Materials and Tissue Engineering, Taipei Medical University, Taipei, Taiwan; 5 Department of Biomedical Engineering, MacKay Memorial Hospital, Taipei, Taiwan; 6 International Ph.D. Program in Cell Therapy and Regenerative Medicine, College of Medicine, Taipei Medical University, Taipei, Taiwan; 7 Department of Pulmonology and Respiratory Medicine, Faculty of Medicine, Universitas Indonesia, Jakarta, Indonesia; 8 International Ph.D. Program in Biomedical Engineering, College of Biomedical Engineering, Taipei Medical University, New Taipei City, Taiwan; Versiti Blood Research Institute, UNITED STATES OF AMERICA

## Abstract

Preclinical thoracic micro-computed tomography (micro-CT) is constrained by the brief pulmonary enhancement window of intravenously (IV) administered iodinated contrast, which the kidneys clear rapidly. We performed a feasibility study in mice comparing IV tail-vein injection with intratracheal microspray instillation of the clinical agent iohexol. Mice received iohexol (100 µL, 500 mg/mL) by either route. Whole-body projections were acquired to 120 min and thoracic sections to 30 min, each animal scanned before contrast in the same session. Contrast distribution was recorded by compartment and quantified from regions of interest. IV delivery produced vascular and cardiac opacification with progressive bladder accumulation, which was the largest signal in every IV animal by 120 min. Intratracheal delivery produced widespread, comparatively uniform lung-parenchymal opacification at 5, 15 and 30 min with no discrete bladder opacity at any time; coronal thoracic signal was 0.77 at 5 min and 0.72 at 30 min, against 0.09 to 0.19 after IV injection. Acute safety was assessed at 5 and 29 h by serum biochemistry and histopathology against untreated controls. ALT, AST and blood urea nitrogen did not differ among groups. Creatinine was modestly higher than in controls after both routes, with no difference between routes, consistent with renal excretion of absorbed iohexol. Lung, heart, liver, spleen and kidney showed preserved architecture without acute injury. These data identify intratracheal microspray delivery as a practical way to hold iodine in the lung parenchyma across a thoracic micro-CT acquisition and provide the basis for a confirmatory quantitative study.

## Introduction

Lung cancer remains the leading cause of cancer-related mortality worldwide, accounting for nearly one in five cancer deaths [1,2]. Despite advances in systemic therapy and immunotherapy, prognosis depends heavily on the stage at diagnosis; early-stage disease offers substantially better survival, yet most patients present with advanced tumours because early disease is typically asymptomatic [1]. Improving the sensitivity and accessibility of diagnostic imaging is therefore central to reducing mortality.

Computed tomography (CT) is the cornerstone of thoracic imaging and the modality of choice for lung-cancer detection and surveillance [3]. Landmark trials, including the National Lung Screening Trial and the Dutch and Belgian NELSON study, showed that low-dose CT screening in high-risk populations reduces lung-cancer mortality by 20% to 24% through early detection of small, often sub-centimetre, nodules [3,4]. However, native CT has limited soft-tissue contrast, particularly for lesions adjacent to vascular or airway structures [5], so iodinated contrast agents are routinely used to improve lesion conspicuity and vascular delineation. In the preclinical setting, micro-CT extends these principles to small-animal models and is an essential tool for longitudinal studies of pulmonary pathology, drug response, and imaging-agent development [6,7]. Because micro-CT acquisitions in small animals take many minutes, the imaging window available with a rapidly cleared IV agent is particularly restrictive [8].

IV administration of nonionic iodinated agents such as iohexol is the standard approach for contrast-enhanced CT. Iohexol is a low-osmolality monomeric agent that is widely used because of its rapid clearance and relatively low rate of adverse effects compared with high-osmolality agents [9,10]. Its use nevertheless has practical limitations. First, IV iohexol has a short circulation half-life and undergoes rapid renal excretion, which constrains the window for pulmonary enhancement and is especially limiting for the long acquisition times of preclinical micro-CT [8,10]. Second, systemic delivery produces non-specific biodistribution, with contrast concentrating in highly perfused organs such as the kidneys and bladder rather than in the lungs [9,10]. Third, iohexol has been associated with contrast-induced nephropathy, particularly in patients with pre-existing renal impairment, and hypersensitivity reactions remain a clinical concern [10,11]. Strategies to extend the pulmonary contrast window include long-circulating nanoparticle agents [12,13] and, as explored here, redirecting an established clinical agent to the airway compartment.

Targeted delivery of contrast directly to the lungs has attracted increasing attention. Pulmonary-route administration deposits the contrast medium into the bronchiolar and alveolar spaces, increasing local concentration and reducing systemic exposure. Preclinical studies have shown that aerosolized iodine-based agents can enhance pulmonary contrast on CT and support functional lung imaging such as regional ventilation mapping [14,15]. Zhang and colleagues reported marked lung enhancement after aerosolized iodinated contrast inhalation in rabbits, with dual-energy CT enabling visualization of functional lobar anatomy [16]. Synchrotron CT imaging in rabbits has likewise shown that aerosol-based delivery can reveal regional ventilation and deposition patterns at high spatial resolution [17].

Few studies have specifically evaluated iohexol for pulmonary delivery. Kang and colleagues used iohexol during video-fluoroscopic swallow studies, in which small quantities were aspirated into the airway, but that application was not intended for pulmonary imaging [18]. A direct comparison between IV and airway-delivered iohexol with regard to image enhancement, biodistribution, and acute organ toxicity is lacking, and reported concerns about pulmonary toxicity from intratracheal iodine-based agents underscore the need for controlled dosing and safety assessment [14,16].

The aim of this study was to compare pulmonary imaging and acute systemic safety between two delivery routes for iohexol in mice: systemic IV injection and intratracheal microspray instillation with a Penn-Century MicroSprayer (Fig 1). Longitudinal micro-CT was used to establish where contrast from each route distributed within the thorax and how long it remained there, and systemic safety was assessed by serum biochemistry and histopathology of major organs. This work explores whether redirecting an established iodinated contrast agent to the airway could extend the pulmonary imaging window of preclinical micro-CT without unacceptable acute toxicity, as a feasibility comparison and the basis for a subsequent quantitative study.

**Fig 1:**
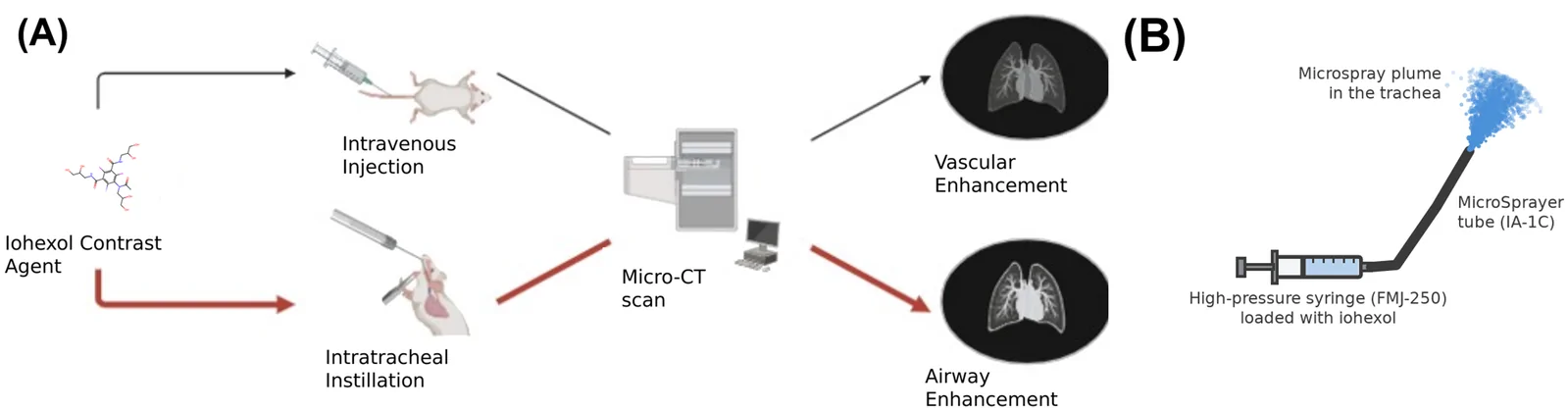
Experimental design and contrast delivery routes. (A) Iohexol was administered by intravenous tail-vein injection (black arrows) or by intratracheal microspray instillation (red arrows), followed by whole-body micro-CT. (B) Intratracheal delivery set-up: a high-pressure syringe drives iohexol through the MicroSprayer tube, which releases a microspray plume at its tip.

## Materials and methods

### Ethics statement

All animal experiments were approved by the Institutional Animal Care and Use Committee (IACUC) of Taipei Medical University (approval numbers LAC-2015–0101 and LAC-2024–0082) and were performed in accordance with applicable national and institutional guidelines for the care and use of laboratory animals. All invasive procedures, including contrast administration, anesthesia, and imaging, were carried out under anesthesia to minimize pain and distress. Animal health and behaviour were monitored throughout the procedures, and a humane endpoint of euthanasia under deep anesthesia was applied at the predefined sampling times. At the end of each experiment, mice were euthanized by exsanguination via cardiac puncture under deep anesthesia, after which organs were harvested. This study is reported in accordance with the ARRIVE 2.0 guidelines for animal research, and a completed checklist is provided as S1 Checklist.

### Contrast-agent solution

Iohexol powder (Histodenz, C_19_H_26_I_3_N_3_O_9_; Sigma-Aldrich, MO, USA) was dissolved in phosphate-buffered saline (PBS; Gibco, NY, USA) at 500 mg/mL. A total volume of 100 µL of iohexol solution per mouse was used for in vivo testing.

### Animals, group allocation, and contrast delivery

Male SCID mice (8–10 weeks old; approximately 25 g) were used for all in vivo experiments, with four animals per group in the imaging analysis and five animals per group per time point in the serum-biochemistry analysis. Mice were maintained under specific-pathogen-free conditions with autoclaved chow and water. The SCID strain was used because these experiments were performed within a broader lung-cancer xenograft imaging program that requires immunodeficient hosts; tumour burden was not a study variable. Male mice were used to reduce experimental variability arising from the estrous cycle and hormonal fluctuation in female mice.

Anesthesia was induced by intraperitoneal injection of 100 µL of a mixture of Zoletil 50 (Virbac, France) and 2% Rompun (Bayer, Korea) combined 1:1 and diluted 6-fold in PBS. Animals were allocated to the IV or intratracheal route and to the 5- and 29-h sampling time points.

For intratracheal microspray delivery, anesthetized mice were fixed on a holder and the trachea was visualized with a laryngoscope (RW-A3748, Hallowell EMC, MA, USA). A Penn-Century MicroSprayer Aerosolizer (Model IA-1C, PA, USA) connected to a high-pressure syringe (FMJ-250) was inserted into the trachea, and the contrast agent was delivered as two sequential 50-µL sprays (total 100 µL of 500 mg/mL iohexol; 50 mg iohexol, approximately 23.2 mg iodine per mouse, or approximately 928 mg iodine per kg body weight [mg I/kg]). The same volume and dose were administered by tail-vein IV injection in the comparator group.

### Micro-computed tomography

Micro-CT imaging was performed on a SkyScan 1076 system (Bruker, Kontich, Belgium) at the National Laboratory Animal Center (Nangang, Taipei). Scanning parameters were: tube voltage 80 kV, current 200 µA, 0.5-mm aluminium filter, voxel resolution 35 µm, and 0.225 s per frame. Each animal was scanned before contrast administration and at 5, 15, 30, 60, and 120 min after administration in the same session and with the same parameters, under continuous anesthesia with spontaneous breathing. Reconstructed volumes were visualized with CT-Analyser software (Bruker), and pseudocolour overlays were generated for comparison of thoracic contrast distribution between groups. Image acquisition was performed by a facility technician who was unaware of the group allocation.

### Image assessment

Regions of interest (ROIs) were placed on the urinary bladder and the lung field in the whole-body projections, and on the central thoracic region in the coronal and axial sections, at every time point. The mean gray-level intensity of each ROI was extracted in Python 3. For each animal, the pre-contrast value was subtracted from every post-contrast value and the results were normalized to that animal’s highest value across all ROIs and time points, giving normalized relative signal in arbitrary units (a.u.), 0 at baseline and 1 at each animal’s maximum. Quantification was performed independently by two readers, a pulmonologist blinded to group and time point and a micro-CT-trained investigator who was not blinded; the two sets of measurements were concordant and were averaged for analysis. Values are presented as the mean and range of four animals per group.

### Serum biochemistry and histology

After contrast administration, mice were euthanized as described above at 5 and 29 h. Blood was collected directly from the heart without anticoagulant, allowed to clot at room temperature for 30 min, and centrifuged at 1500 × g for 15 min at 4 °C to separate serum. Serum was analyzed on a biochemical analyzer (Catalyst One; IDEXX Laboratories, ME, USA) for hepatic markers (alanine aminotransferase, ALT; aspartate aminotransferase, AST) and renal markers (blood urea nitrogen, BUN; creatinine, CREA). Untreated mice served as normal controls. For histology, the lung, heart, liver, spleen, and kidney were excised immediately after euthanasia, fixed in 10% neutral-buffered formalin for at least 24 h, processed for paraffin sectioning, stained with hematoxylin and eosin (H&E), and examined under a light microscope (Nikon Eclipse Ei, Tokyo, Japan).

### Statistical analysis

The imaging comparison is descriptive: normalized relative signal is summarized as the mean and range of four animals per group. For serum biochemistry, each analyte was analyzed by two-way ANOVA with administration group (untreated control, IV, intratracheal) and time point (5 and 29 h) as fixed factors, followed by Tukey’s post hoc test comparing the three groups within each time point. Variance homogeneity was assessed by inspection of the per-cell standard deviations (S1 Data). All tests were two-sided, and an adjusted p < 0.05 was considered significant; no correction was applied across the four analytes. Statistical analysis and graphs were prepared in GraphPad Prism 10 (GraphPad Software, Boston, MA, USA). Summary statistics are given in S1 Data.

## Results

### Biodistribution and systemic clearance of iohexol

Whole-body micro-CT projections were acquired at baseline and up to 120 min after administration (Fig 2A). After IV injection, contrast distributed systemically almost immediately; by 5 min, a concentrated signal was present in the urinary bladder, consistent with rapid renal clearance. Normalized bladder enhancement in the IV group rose from 0.54 a.u. (range 0.48 to 0.59) at 5 min to 0.77 a.u. at 15 min and reached 1.00 a.u. at 120 min, the bladder at 120 min being each animal’s single largest reading (Fig 2B).

**Fig 2:**
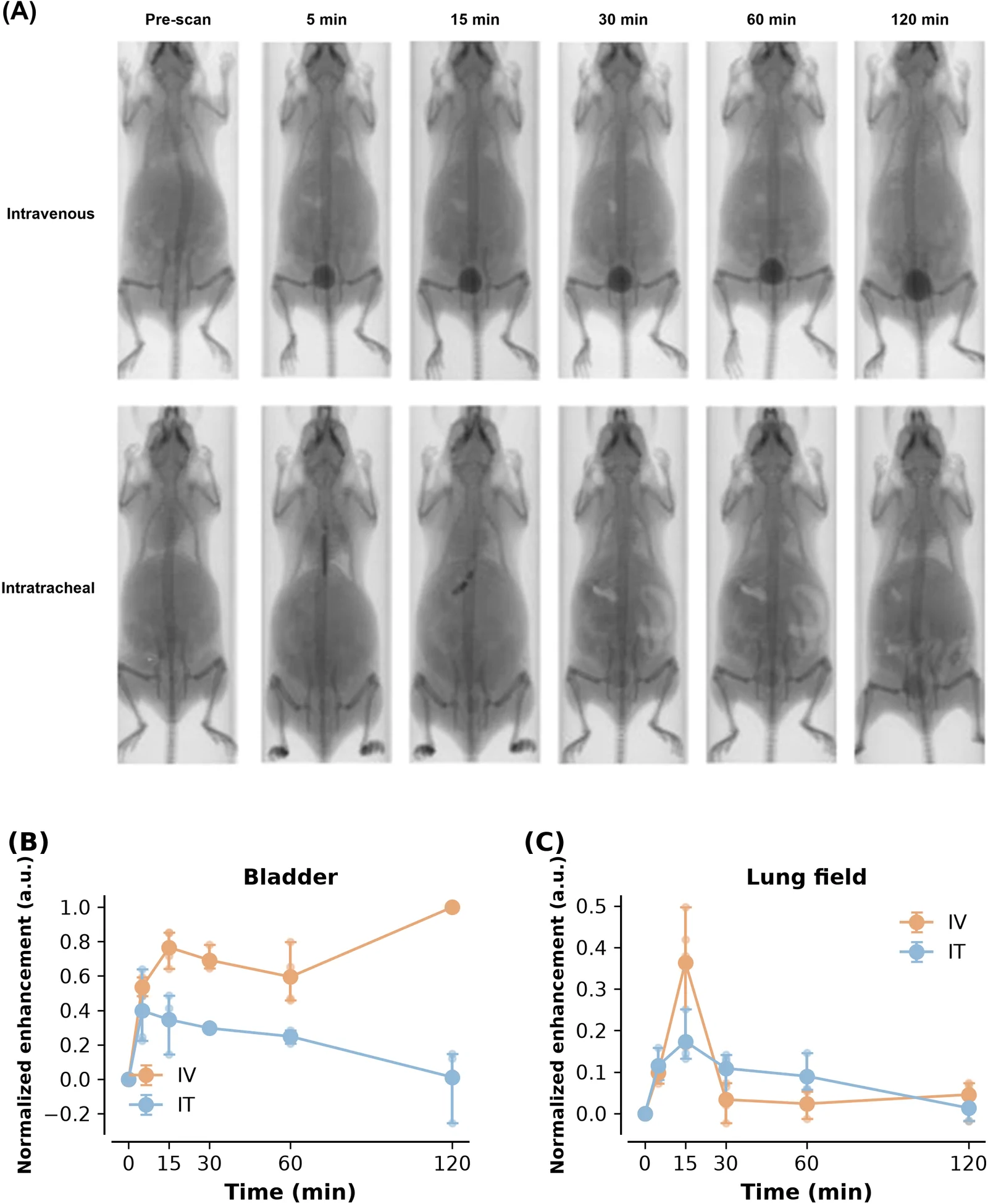
Whole-body micro-CT of iohexol biodistribution after intravenous or intratracheal administration. (A) Representative whole-body projections before contrast and at 5, 15, 30, 60 and 120 min after administration. (B, C) Region-of-interest quantification of the bladder (B) and the lung field (C). The x axis is time after administration (min); the y axis is normalized enhancement in arbitrary units (a.u.), 0 at each animal’s pre-contrast baseline and 1 at its maximum. Large symbols show the group mean, bars the range, and small symbols individual animals (n = 4 per group). IV, intravenous; IT, intratracheal.

Intratracheal microspray delivery localized the contrast agent within the thoracic cavity, and no discrete bladder opacity of the kind seen after IV injection was apparent at any time point (Fig 2A). The bladder ROI after instillation was highest at 5 min (0.40 a.u., range 0.22 to 0.64), declined to 0.25 a.u. by 60 min and was at baseline at 120 min (0.01 a.u.). The bladder measurement therefore rose progressively after IV injection and declined to baseline after instillation.

The lung-field ROI on the projections did not exceed a group mean of 0.36 a.u. on either route and returned to within 0.05 a.u. of baseline by 120 min (Fig 2C). From 30 to 60 min the intratracheal group retained a higher lung-field signal (0.11 and 0.09 a.u.) than the IV group (0.03 and 0.02 a.u.), which had returned to near baseline by 30 min. Projections are insensitive to parenchymal change because of overlying bone and heart; the tomographic sections (Fig 3) provide the parenchymal information.

**Fig 3:**
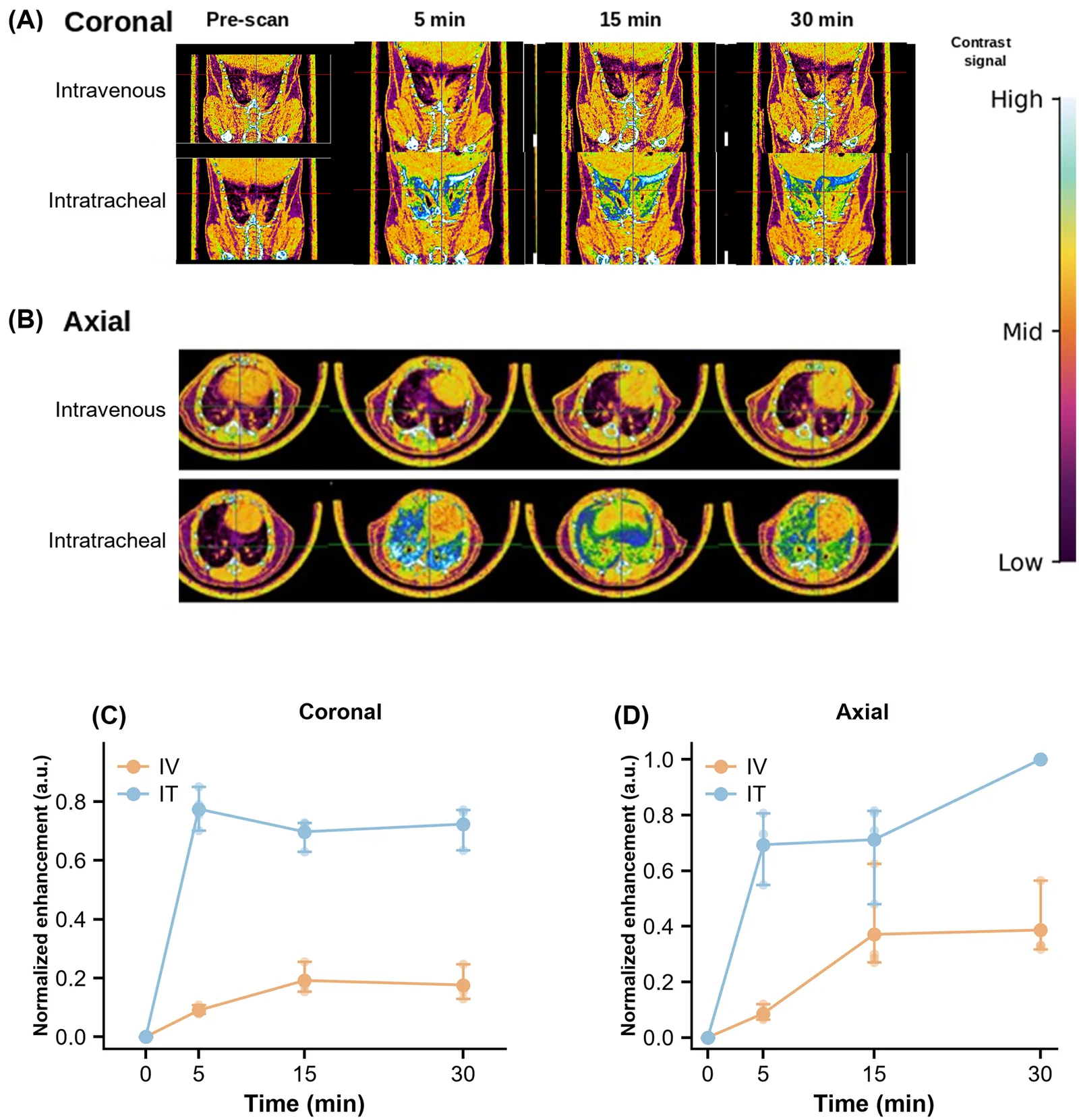
Pulmonary contrast enhancement on thoracic micro-CT sections. (A) Coronal and (B) axial sections before contrast and at 5, 15 and 30 min after intravenous or intratracheal administration, displayed with a pseudocolour scale from low (dark) to high (white) contrast signal; representative of four animals per group. (C, D) Normalized enhancement within a central thoracic region of interest on the coronal (C) and axial (D) sections. The x axis is time after administration (min); the y axis is normalized enhancement in arbitrary units (a.u.), 0 at each animal’s pre-contrast baseline and 1 at its maximum. Large symbols show the group mean, bars the range, and small symbols individual animals (n = 4 per group). IV, intravenous; IT, intratracheal.

### Pulmonary contrast enhancement

Pseudocolour thoracic reconstructions show the divergent contrast-distribution patterns directly (Fig 3). In the IV group, enhancement on both coronal (Fig 3A) and axial (Fig 3B) views was confined mainly to the central vasculature and heart, and the lung fields changed little from the pre-contrast appearance at any of the three post-contrast time points. In the intratracheal microspray group, enhancement appeared within the lung parenchyma itself, was already present at 5 min, and remained comparatively uniform across both lungs at 15 and 30 min. The difference between the routes is therefore one of anatomical distribution as well as of magnitude.

Region-of-interest quantification follows the images. In the coronal view (Fig 3C), normalized signal in the intratracheal group was 0.77 a.u. (range 0.70 to 0.85) at 5 min, 0.70 a.u. (0.63 to 0.73) at 15 min and 0.72 a.u. (0.64 to 0.77) at 30 min, against 0.09 a.u. (0.08 to 0.11), 0.19 a.u. (0.15 to 0.26) and 0.18 a.u. (0.13 to 0.25) for the IV group. In the axial view (Fig 3D), the intratracheal group was 0.69 a.u. (0.55 to 0.81) at 5 min, 0.71 a.u. (0.48 to 0.82) at 15 min and 1.00 a.u. at 30 min in every animal, against 0.09 a.u. (0.07 to 0.12), 0.37 a.u. (0.27 to 0.63) and 0.39 a.u. (0.32 to 0.56) for IV. The IV axial curve rose across the three time points because the central thoracic ROI contains the heart and great vessels as well as lung. Neither IV curve approached the intratracheal values at any time point, and the intratracheal signal present at 5 min was still present, without decline, at 30 min.

### Systemic safety and histology

Hepatic and renal serum markers were measured at 5 and 29 h after administration and analyzed by two-way ANOVA with Tukey comparisons (Fig 4). ALT (Fig 4A), AST (Fig 4B), and BUN (Fig 4C) showed no significant effect of administration group. All three were lower at 29 h than at 5 h (effect of time, p < 0.01), with no group-by-time interaction.

**Fig 4:**
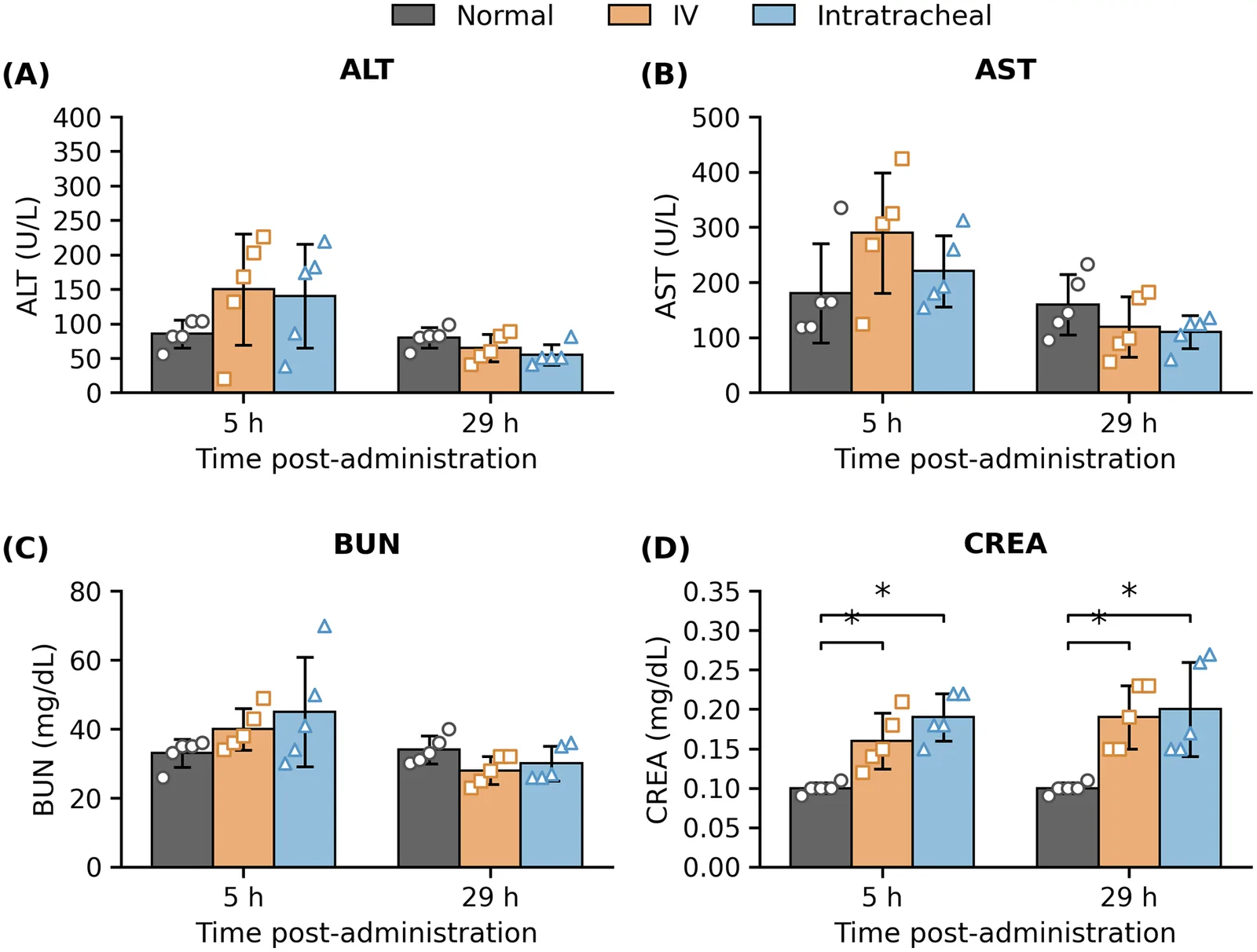
Serum biochemistry at 5 and 29 h after administration by different routes. (A) ALT, (B) AST, (C) BUN and (D) CREA in untreated control (Normal), intravenous (IV) and intratracheal groups. The x axis is time after administration; the y axis is serum concentration (U/L for ALT and AST, mg/dL for BUN and CREA). Bars show the group mean with standard deviation, and each symbol is an individual animal. *Adjusted p < 0.05 versus untreated control.

For creatinine (Fig 4D), the group effect was significant (p < 0.001), with no effect of time or interaction. Against an untreated control mean of 0.10 mg/dL, creatinine was higher after both routes: 0.16 mg/dL (IV) and 0.19 mg/dL (intratracheal) at 5 h, and 0.19 and 0.20 mg/dL at 29 h (all adjusted p < 0.05 versus control). The two routes did not differ from each other at either time point. This small, route-independent increase is consistent with renal excretion of absorbed iohexol; BUN and renal histology were unchanged (Fig 5), and as the only significant result among four analytes tested without cross-analyte correction it is regarded as an exploratory observation. Intratracheal delivery was therefore at least as well tolerated as IV injection on this acute biomarker panel.

**Fig 5:**
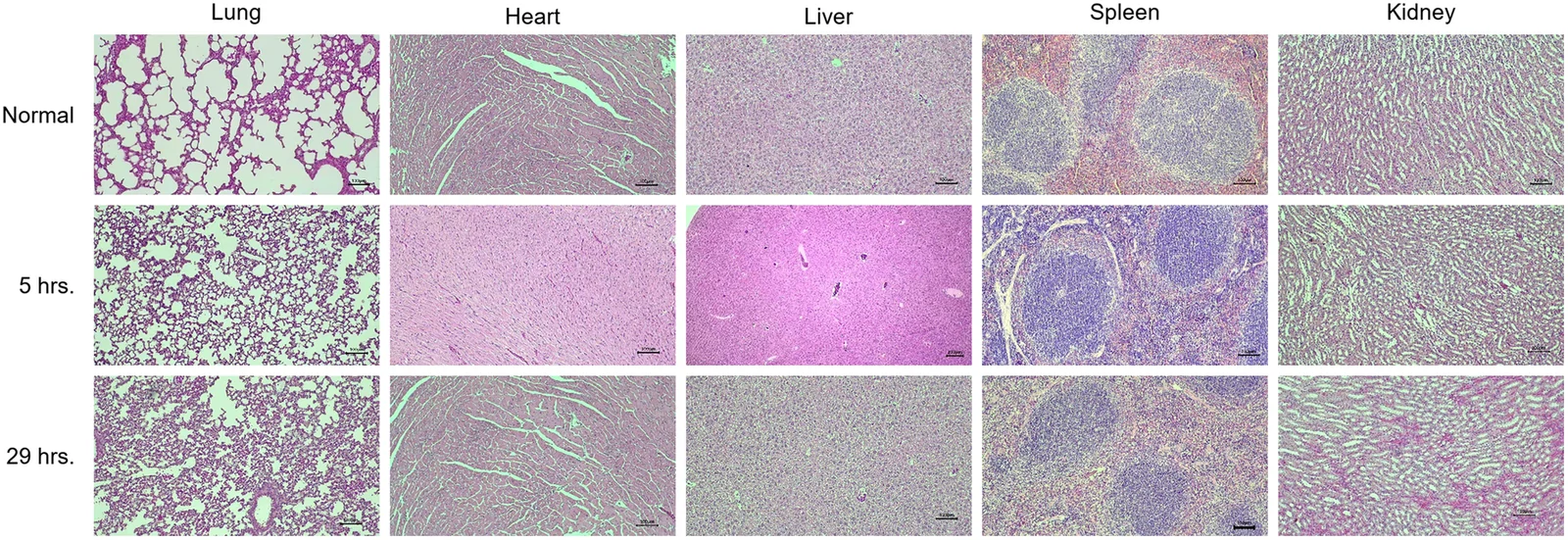
Histological sections from normal mice and from mice given intratracheal iohexol. Representative H&E-stained sections of lung, heart, liver, spleen and kidney from untreated control animals (Normal) and from animals at 5 and 29 h after intratracheal microspray administration. Scale bars, 100 µm (200 µm in the liver and kidney panels at 5 h).

Representative H&E sections from the intratracheal group and from normal mice are shown in Fig 5. The lung preserved normal alveolar architecture with intact septa and clear airspaces at 5 and 29 h; no inflammatory infiltrates, interstitial edema, or alveolar hemorrhage were observed, and the bronchiolar epithelium was intact. Heart, liver, spleen, and kidney sections were similarly unremarkable. Together with the serum biochemistry, these findings indicate no detectable acute organ toxicity at the examined time points in the intratracheal iohexol-treated mice.

## Discussion

This feasibility study compared intratracheal microspray instillation and conventional IV administration of iohexol for thoracic micro-CT. The two routes did not differ merely in degree; they filled different anatomical compartments and behaved differently over time. The airway route held iodine in the lung parenchyma throughout the 30-min tomographic window, while IV injection produced rapid vascular filling followed by renal clearance, with bladder signal rising to become each animal’s largest reading by 120 min. No acute systemic toxicity was observed on serum biomarkers or histopathology of five organs. For a preclinical imaging workflow, a 30-min enhancement window is sufficient for a complete acquisition [6–8].

The divergent imaging outcomes reflect different pharmacokinetics. After IV injection, iohexol fills the vascular compartment and is rapidly cleared by glomerular filtration, with a distribution half-life of minutes and an elimination half-life of approximately 2 to 4 h in subjects with normal renal function [19]. This rapid clearance accounts for the transient vascular opacification seen clinically and necessitates precisely timed scan protocols to capture peak enhancement [20,21]. Our data recapitulate this pattern: contrast appeared in the bladder within 5 min of IV injection (Fig 2), the renal route that underlies the clinical concern about contrast-associated kidney injury [22,23]. This rapid washout is a well-recognized constraint of preclinical micro-CT and has motivated the development of long-circulating iodinated agents [12,13].

The Penn-Century MicroSprayer produces a bolus liquid spray rather than a fine respirable mist. Cossío and colleagues reported that it generates droplets of approximately 20 µm and achieves more than 85% pulmonary deposition, compared with as little as 0.1% for nebulizer-based systems [24]. This high-efficiency delivery is consistent with the comparatively uniform parenchymal distribution in our micro-CT reconstructions (Fig 3). Anesthesia and spontaneous breathing during micro-CT can shift regional aerosol distribution [25], a known confounder mitigated through protocol standardization [26,27].

A strength of this study is the use of a clinically available contrast agent on a conventional micro-CT platform, providing a direct preclinical analogue of clinical CT. The design is internally controlled: every animal was scanned before contrast in the same session, so enhancement is judged against each animal’s own baseline. Extending the effective enhancement window has practical value for longitudinal studies of pulmonary disease progression and therapeutic response [8,12]. This approach is complementary to long-circulating blood-pool agents (liposomal and nanoparticle formulations), which prolong enhancement of the pulmonary vasculature rather than the airspace and therefore address different imaging questions, such as perfusion rather than parenchymal distribution [28]. Its advantages are that the agent, dose range and safety profile are already established clinically and no formulation development is required; its limitation is that clearance from the airspace sets the imaging window, which here comfortably exceeded the 30-min tomographic series. Although synchrotron CT has imaged aerosol deposition at very high spatiotemporal resolution [17], the present study validates the concept with tools and agents already available in standard preclinical imaging environments.

Intratracheal microspray deposits iohexol on airway and alveolar surfaces at high local concentration, favouring diffusion into peribronchiolar fluid and the distal acinus. In small rodents, the MicroSprayer favours distal airway and alveolar delivery [24], and prior studies of airway-delivered iodinated contrast have shown broad parenchymal coverage with prolonged residence relative to IV delivery [17,29]. Our coronal and axial reconstructions showed comparatively uniform signal across both lung fields at 5, 15 and 30 min (Fig 3), consistent with iohexol occupying the epithelial lining fluid and small-airway lumen before clearance by mucociliary transport and macrophage uptake [25,30]. Beyond 30 min only whole-body projections are available, which are insensitive to parenchymal change; a tomographic time course extending to 120 min is the next step to define the duration of parenchymal persistence.

Safety is a central consideration for airway-delivered contrast. Zhang and colleagues demonstrated aerosolized iodinated contrast in rabbits for dual-energy CT ventilation imaging but did not include a detailed safety assessment [16]. The present study addresses this gap. The administered dose (approximately 928 mg iodine per kg) is comparable in order of magnitude to routine clinical IV loads (approximately 430–750 mg iodine per kg) [21], though direct scaling is confounded by allometric differences and the local versus systemic route. ALT, AST, and BUN did not differ among groups, and histology showed no acute injury at either time point (Figs 4 and 5). Serum creatinine rose modestly after both routes, with no difference between routes. Iohexol is eliminated unchanged by glomerular filtration and is itself used as an exogenous marker of glomerular filtration rate [19]; iohexol deposited in the airspace is absorbed into the circulation and excreted by the same path, consistent with the early bladder signal after instillation (Fig 2B), which explains why the two routes behaved alike. This is consistent with recent evidence that IV iodinated contrast is less nephrotoxic than historically believed [22,31–33]. The increase (0.06 to 0.10 mg/dL) is well below the 0.3 mg/dL rise that defines post-contrast acute kidney injury [22,32], the values sit near the lower reportable limit of the analyzer (0.1 mg/dL), and BUN and renal histology were unchanged, so we interpret it as a transient functional response to renal excretion of the agent rather than injury. The safety data cover acute systemic tolerance; local pulmonary inflammatory responses, lung mechanics, mucociliary clearance and repeat-dosing effects were not assessed, and bronchoalveolar lavage and lung-function endpoints are planned for the confirmatory study.

Animals were assigned to route in the order in which they became available, and each animal served as its own pre-contrast control. Image acquisition was performed by a technician unaware of group allocation, and quantification was performed independently by a blinded and a non-blinded reader with concordant results. The primary difference between routes is categorical, which compartment contains contrast, rather than graded, which leaves little room for reader bias. Randomized allocation and fully blinded scoring are planned for the confirmatory study.

This feasibility study has limitations. Attenuation is reported as normalized relative signal rather than in Hounsfield units because no density calibration phantom was included in the field of view, so the imaging comparison is descriptive. Group sizes were small (n = 4 per group for imaging, n = 5 for serum) and were not set by a power calculation; the study was designed to establish feasibility and the direction of effect, which it does, and the confirmatory study should be powered from calibrated measurements. SCID mice lack adaptive immunity, so hypersensitivity reactions to iodinated contrast cannot be evaluated in this strain, and only male mice were used. Intratracheal microspray instillation is not equivalent to clinical nebulized inhalation; translation will require nebulizer-based or dry-powder systems [24,25]. The confirmatory study should include a density calibration phantom for Hounsfield-unit quantification, tomographic sampling to 120 min, randomized allocation with blinded reading, immunocompetent animals of both sexes, and bronchoalveolar lavage and lung-function endpoints.

## Conclusions

Intratracheal microspray delivery of iohexol is technically feasible in mice and was acutely well tolerated at the dose and time points examined. IV injection produced contrast in the central vessels and bladder within 5 min, whereas intratracheal instillation produced bilateral lung-parenchymal opacification that persisted throughout the 30-min tomographic series with no discrete bladder opacity. Serum biochemistry and histopathology of five organs showed no acute toxicity; the small creatinine elevation was route-independent and consistent with renal excretion of the agent. A powered, quantitative, blinded study in immunocompetent animals of both sexes, acquired with a density calibration phantom, is the logical next step to quantify the attenuation difference and to define the duration of parenchymal persistence beyond 30 min. These data establish that the approach works with an agent and platform already in routine use.

## Supporting information

S1 Checklist ARRIVE 2.0 author checklist. (DOCX)

S1 Data Image quantification (individual animal values) and serum-biochemistry summary statistics. (XLSX)
