# Supplementary material for: Intratracheal microspray delivery of iohexol for sustained lung-parenchymal enhancement in preclinical micro-computed tomography: A feasibility and acute-safety study in mice
Source: PLoS One. 2026 Oct 23;21(10):e0361103. doi: 10.1371/journal.pone.0361103 (PMC13693886; doi:10.1371/journal.pone.0361103)
Supplement: Supplementary file 1 [file pone.0361103.s001.docx]

**S1 Checklist. ARRIVE 2.0 author checklist**

Intratracheal microspray delivery of iohexol for sustained lung-parenchymal enhancement in preclinical micro-computed tomography: a feasibility and acute-safety study in mice.

Completed against the ARRIVE 2.0 guidelines (Percie du Sert et al., PLoS Biol. 2020;18(7):e3000410). Section references point to the accompanying manuscript.

**The ARRIVE Essential 10**

| Item | How and where addressed |
| --- | --- |
| 1. Study design | Feasibility study: IV versus intratracheal iohexol, plus untreated controls. (Methods.) |
| 2. Sample size | n = 4 per group for imaging, 5 per group per time point for serum biochemistry; no power calculation; feasibility study. (Methods: Animals; Statistical analysis; Discussion.) |
| 3. Inclusion and exclusion criteria | No predefined inclusion or exclusion criteria were applied and no animals or data points were excluded from analysis. |
| 4. Randomization | Animals assigned to route in order of availability; each animal served as its own pre-contrast control. (Methods: Animals; Discussion.) |
| 5. Blinding | Micro-CT acquisition by a facility technician unaware of group allocation. Image quantification by two independent readers, one blinded (pulmonologist) and one non-blinded (micro-CT-trained investigator); concordant results averaged for analysis. (Methods: Micro-computed tomography; Image assessment; Discussion.) |
| 6. Outcome measures | Primary: thoracic contrast distribution by compartment and ROI-based normalized relative signal over time (n = 4). Secondary: serum ALT, AST, BUN, creatinine; histopathology of five organs. (Methods.) |
| 7. Statistical methods | Two-way ANOVA with Tukey post hoc (adjusted p < 0.05) in GraphPad Prism 10; variance homogeneity assessed by inspection of per-cell SDs. Imaging: descriptive (mean and range); ROI intensities extracted in Python 3. (Methods: Image assessment; Statistical analysis.) |
| 8. Experimental animals | Male SCID mice, 8 to 10 weeks, approximately 25 g, SPF housing. Strain rationale and male-only cohort explained. (Methods: Animals.) |
| 9. Experimental procedures | Zoletil/Rompun anesthesia; 100 µL iohexol (500 mg/mL) IV or intratracheal via MicroSprayer; SkyScan 1076 micro-CT (80 kV, 35 µm); cardiac-puncture euthanasia. (Methods.) |
| 10. Results | Group-level data with variability in Figs 2 to 5; individual imaging values and serum summary statistics in S1 Data. (Results; Figs 2 to 5.) |

**The Recommended Set**

| Item | How and where addressed |
| --- | --- |
| 11. Abstract | Species, methods, results and feasibility scope stated. (Abstract.) |
| 12. Background | Rationale for airway-route contrast delivery to extend the pulmonary micro-CT enhancement window. (Introduction.) |
| 13. Objectives | To compare pulmonary imaging and acute systemic safety of IV versus intratracheal iohexol as a feasibility study. (Introduction, final paragraph.) |
| 14. Ethical statement | Approved by the Taipei Medical University IACUC (LAC-2015-0101, LAC-2024-0082); conducted under applicable national and institutional guidelines. (Methods: Ethics statement.) |
| 15. Housing and husbandry | Specific-pathogen-free housing with autoclaved chow and water. (Methods: Animals.) |
| 16. Animal care and monitoring | All invasive procedures performed under anesthesia; health and behaviour monitored throughout; humane endpoint of euthanasia under deep anesthesia applied at predefined sampling times. (Methods: Ethics statement.) |
| 17. Interpretation and scientific implications | Conclusions confined to feasibility; imaging comparison descriptive in normalized relative units. (Discussion; Conclusions.) |
| 18. Generalizability and translation | Limitations stated for imaging scale, cohort size, safety scope, strain, sex, and delivery method, with the confirmatory study design. (Discussion, final paragraph.) |
| 19. Protocol registration | The study protocol was not pre-registered (preclinical feasibility study). |
| 20. Data access | Individual per-animal values for Figs 2 and 3 and summary statistics for Fig 4 in S1 Data. (S1 Data; Data Availability.) |
| 21. Declaration of interests | No competing interests. Declared in submission fields. (Acknowledgments.) |
