# Supplementary material for: Intratracheal microspray delivery of iohexol for sustained lung-parenchymal enhancement in preclinical micro-computed tomography: A feasibility and acute-safety study in mice
Source: PLoS One. 2026 Oct 23;21(10):e0361103. doi: 10.1371/journal.pone.0361103 (PMC13693886; doi:10.1371/journal.pone.0361103)
Supplement: Supplementary file 2 [file pone.0361103.s004.docx]

**Responses to Reviewers’ Comments**

We have responded to the reviewers’ comments one by one below. All modifications made in response to the comments are marked in blue in the revised manuscript, with their location given as section, page and line.

**Academic Editor Comments:**

**Dr. Kaminski highlighted the need for quantitative support of the imaging findings, clearer emphasis on the exploratory nature of the study, and more explicit discussion of limitations related to pulmonary safety, potential bias, and the use of male SCID mice.**

**Response:** We thank the Editor for this comment. Image quantification has been done and the results are shown in Figs 2 and 3 of the revised manuscript, with the related description in the Methods, Results and Discussion. The feasibility scope of the study is stated in the Abstract, Introduction, Discussion and Conclusions, and the Discussion now covers pulmonary safety, allocation and blinding, and the SCID strain and male-only cohort. Details are given under each reviewer comment below.

**Journal Requirements:**

**1. Please ensure that your manuscript meets PLOS ONE’s style requirements, including those for file naming.**

**Response:** Thank you for this reminder. The manuscript follows the PLOS ONE templates for the main body and the title page. Headings follow the required order, figures are cited as “Fig N”, and references follow the Vancouver style.

**2. We note that the grant information you provided in the ‘Funding Information’ and ‘Financial Disclosure’ sections do not match.**

**Response:** The Funding Information and Financial Disclosure sections have been reconciled so that the grant numbers match exactly.

**3. If the reviewer comments include a recommendation to cite specific previously published works, please review and evaluate these publications.**

**Response:** We have reviewed the relevant literature and incorporated the references suited to this manuscript (Reviewer #1, Minor comment 1).

**Reviewer #1:**

Major Comments

**1. The primary imaging outcome is evaluated only qualitatively. Quantitative image analysis (e.g., ROI-based Hounsfield unit measurements, time-density curves, or signal intensity comparisons) is not provided.**

**Response:** We agree that quantitative measurements strengthen the conclusion and have added them. Regions of interest were placed on the bladder and lung field in the whole-body projections (Fig 2B, 2C) and on the central thoracic region in the coronal and axial sections (Fig 3C, 3D) at every time point, for four animals per group. Because no density calibration phantom was scanned, signal is expressed as normalized relative signal in arbitrary units (a.u.), with each animal’s pre-contrast scan as its own baseline; this is the signal-intensity comparison suggested by the Reviewer. The quantification confirms the images: intratracheal delivery gave a coronal thoracic signal of 0.77 a.u. at 5 min and 0.72 a.u. at 30 min, against 0.09 to 0.19 a.u. after IV injection. All individual values are provided in S1 Data.

*“Regions of interest (ROIs) were placed on the urinary bladder and the lung field in the whole-body projections, and on the central thoracic region in the coronal and axial sections, at every time point. The mean gray-level intensity of each ROI was extracted in Python 3.”* **(Materials and methods, Image assessment, page 8, lines 150-152)**

*“In the coronal view (Fig 3C), normalized signal in the intratracheal group was 0.77 a.u. (range 0.70 to 0.85) at 5 min, 0.70 a.u. (0.63 to 0.73) at 15 min and 0.72 a.u. (0.64 to 0.77) at 30 min, against 0.09 a.u. (0.08 to 0.11), 0.19 a.u. (0.15 to 0.26) and 0.18 a.u. (0.13 to 0.25) for the IV group.”* **(Results, Pulmonary contrast enhancement, page 11, lines 216-219)**

**2. The sample size is small and the study should clearly be presented as exploratory. The limited statistical power should be discussed more explicitly, and the findings should be described as hypothesis-generating rather than definitive.**

**Response:** We agree that the scope of the study should be clear. The study is presented throughout as a feasibility comparison, and the findings as the basis for a confirmatory quantitative study. The Discussion states that group sizes were not set by a power calculation and how the confirmatory study should be powered, and the Conclusions name that study as the next step. The direction and location of the effect were consistent in every animal (Figs 2 and 3; S1 Data), which is what a feasibility study is designed to establish.

*“Group sizes were small (n = 4 per group for imaging, n = 5 for serum) and were not set by a power calculation; the study was designed to establish feasibility and the direction of effect, which it does, and the confirmatory study should be powered from calibrated measurements.”* **(Discussion, pages 16 to 17, lines 341-344)**

*“A powered, quantitative, blinded study in immunocompetent animals of both sexes, acquired with a density calibration phantom, is the logical next step to quantify the attenuation difference and to define the duration of parenchymal persistence beyond 30 min.”* **(Conclusions, page 17, lines 359-361)**

**3. The safety assessment is limited to acute systemic toxicity. Local pulmonary inflammatory responses and pulmonary function were not evaluated; this limitation should be more clearly acknowledged in the Discussion.**

**Response:** The Discussion now states what the safety assessment covers, names the pulmonary endpoints not evaluated, and identifies them as endpoints of the confirmatory study.

*“The safety data cover acute systemic tolerance; local pulmonary inflammatory responses, lung mechanics, mucociliary clearance and repeat-dosing effects were not assessed, and bronchoalveolar lavage and lung-function endpoints are planned for the confirmatory study.”* **(Discussion, page 16, lines 329-332)**

Minor Comments

**1. The manuscript would benefit from a more detailed discussion comparing this approach with previously reported long-circulating or nanoparticle-based CT contrast agents, highlighting the relative advantages and limitations of using a clinically available iodinated contrast agent.**

**Response:** We appreciate this suggestion and have expanded the comparison in the Discussion. Blood-pool agents prolong enhancement of the pulmonary vasculature, whereas intratracheal delivery fills the airspace, so the two strategies address different imaging questions and are complementary [1]. The advantages of a clinically available agent (established dose range and safety profile, no formulation development) and its limitation (clearance from the airspace sets the imaging window) are now stated.

*“Its advantages are that the agent, dose range and safety profile are already established clinically and no formulation development is required; its limitation is that clearance from the airspace sets the imaging window, which here comfortably exceeded the 30-min tomographic series.”* **(Discussion, pages 14 to 15, lines 297-300)**

**2. The lack of randomization and blinded image assessment should be acknowledged as potential sources of bias when interpreting the qualitative imaging results.**

**Response:** Thank you for this concern. We have added the procedure to the Methods and Discussion. Animals were assigned to route in the order they became available, and each animal was scanned before contrast in the same session as its own control. The CT examinations were acquired by a facility technician who did not know the group allocation. The images were then quantified by two readers, a pulmonologist blinded to group and time point and a micro-CT-trained investigator who was not blinded; their results were concordant and were averaged for analysis. Randomized allocation and fully blinded scoring are specified for the confirmatory study.

*“Image acquisition was performed by a facility technician who was unaware of the group allocation.”* **(Materials and methods, Micro-computed tomography, page 7, lines 147-148)**

*“Quantification was performed independently by two readers, a pulmonologist blinded to group and time point and a micro-CT-trained investigator who was not blinded; the two sets of measurements were concordant and were averaged for analysis.”* **(Materials and methods, Image assessment, page 8, lines 156-158)**

**3. Because only SCID mice were used, the authors should further emphasize that immune-mediated responses and hypersensitivity reactions cannot be evaluated in this model.**

**Response:** This point is now stated in the Discussion, and the confirmatory study design specifies immunocompetent animals of both sexes.

*“SCID mice lack adaptive immunity, so hypersensitivity reactions to iodinated contrast cannot be evaluated in this strain, and only male mice were used.”* **(Discussion, page 17, lines 344-346)**

**Reviewer #2:**

We are grateful for the positive evaluation and have addressed each suggestion below.

**1. The rationale for using only male animals is stated as a limitation but not explained in the Methods. A one-line clarification would tie this off more completely.**

**Response:** Thank you for this suggestion. A one-line explanation has been added to the Methods.

*“Male mice were used to reduce experimental variability arising from the estrous cycle and hormonal fluctuation in female mice.”* **(Materials and methods, Animals, group allocation, and contrast delivery, page 6, lines 127-128)**

**2. Given the small per-cell sample size, consider briefly noting whether variance homogeneity was checked, since Tukey HSD and the pooled variance estimate assume roughly equal variance across cells.**

**Response:** Variance homogeneity was assessed by inspecting the per-cell standard deviations, which are given in S1 Data, and this is now stated in the Statistical analysis section. The creatinine effect is large relative to the within-cell spread (treated means exceed the control mean by 0.06 to 0.10 mg/dL, against standard deviations of 0.01 to 0.06 mg/dL) and is present at both time points, and the between-route comparison, which involves the two cells with similar spread, was not significant. The conclusions do not depend on the equal-variance assumption.

*“Variance homogeneity was assessed by inspection of the per-cell standard deviations (S1 Data).”* **(Materials and methods, Statistical analysis, page 9, lines 176-177)**

**3. Since creatinine was the only significant finding among four analytes without cross-analyte correction, consider stating explicitly in the Results or Discussion that this finding should be interpreted as exploratory.**

**Response:** We agree. The statement has been added to the Results, and the Discussion now explains the finding. Iohexol is eliminated unchanged by glomerular filtration and is itself used as a marker of glomerular filtration rate [2]. Iohexol deposited in the airspace is absorbed into the circulation and excreted by the same path, which explains why both routes produced the same small increase. The increase (0.06 to 0.10 mg/dL) is well below the 0.3 mg/dL rise that defines post-contrast acute kidney injury [3,4], and BUN and renal histology were unchanged, so it is interpreted as a transient functional response to renal excretion of the agent rather than injury.

*“This small, route-independent increase is consistent with renal excretion of absorbed iohexol; BUN and renal histology were unchanged (Fig 5), and as the only significant result among four analytes tested without cross-analyte correction it is regarded as an exploratory observation.”* **(Results, Systemic safety and histology, page 12, lines 244-247)**

*“Iohexol is eliminated unchanged by glomerular filtration and is itself used as an exogenous marker of glomerular filtration rate [19]; iohexol deposited in the airspace is absorbed into the circulation and excreted by the same path, consistent with the early bladder signal after instillation (Fig 2B), which explains why the two routes behaved alike.”* **(Discussion, pages 15 to 16, lines 320-324)**

**References**

1. Hallouard F, Anton N, Choquet P, Constantinesco A, Vandamme T. Iodinated blood pool contrast media for preclinical X-ray imaging applications: a review. Biomaterials. 2010;31: 6249-6268.

2. Dong Q, Chen Z, Boland J, Dokos C, Hagos Y, Kühne A, et al. Validating low-dose iohexol as a marker for glomerular filtration rate by in vitro and in vivo studies. Clin Transl Sci. 2025;18: e70141.

3. Davenport MS, Perazella MA, Yee J, Dillman JR, Fine D, McDonald RJ, et al. Use of intravenous iodinated contrast media in patients with kidney disease: consensus statements from the American College of Radiology and the National Kidney Foundation. Radiology. 2020;294: 660-668.

4. van der Molen AJ, Reimer P, Dekkers IA, Bongartz G, Bellin MF, Bertolotto M, et al. Post-contrast acute kidney injury, Part 1: definition, clinical features, incidence, role of contrast medium and risk factors. Eur Radiol. 2018;28: 2845-2855.
