# Supplementary material for: Intratracheal microspray delivery of iohexol for sustained lung-parenchymal enhancement in preclinical micro-computed tomography: A feasibility and acute-safety study in mice
Source: PLoS One. 2026 Oct 23;21(10):e0361103. doi: 10.1371/journal.pone.0361103 (PMC13693886; doi:10.1371/journal.pone.0361103)
Supplement: Peer review history

# Peer review: Decision Letter 0

**DOI:** 10.1371/journal.pone.0361103.r001

**Authors:** Tomasz Kaminski

23 Jul 2026

Dear Dr. Tseng,

Thank you for submitting your manuscript to PLOS ONE. After careful consideration, we feel that it has merit but does not fully meet PLOS ONE’s publication criteria as it currently stands. Therefore, we invite you to submit a revised version of the manuscript that addresses the points raised during the review process.

Please submit your revised manuscript by Sep 21 2026 11:59PM. If you will need more time than this to complete your revisions, please reply to this message or contact the journal office at plosone@plos.org. When you're ready to submit your revision, log on to https://www.editorialmanager.com/pone/ and select the 'Submissions Needing Revision' folder to locate your manuscript file.

- A letter that responds to each point raised by the academic editor and reviewer(s). You should upload this letter as a separate file labeled 'Response to Reviewers'.
- A marked-up copy of your manuscript that highlights changes made to the original version. You should upload this as a separate file labeled 'Revised Manuscript with Track Changes'.
- An unmarked version of your revised paper without tracked changes. You should upload this as a separate file labeled 'Manuscript'.

If you would like to make changes to your financial disclosure, please include your updated statement in your cover letter. Guidelines for resubmitting your figure files are available below the reviewer comments at the end of this letter.

If applicable, we recommend that you deposit your laboratory protocols in protocols.io to enhance the reproducibility of your results. Protocols.io assigns your protocol its own identifier (DOI) so that it can be cited independently in the future. For instructions see: https://journals.plos.org/plosone/s/submission-guidelines#loc-laboratory-protocols. Additionally, PLOS ONE offers an option for publishing peer-reviewed Lab Protocol articles, which describe protocols hosted on protocols.io. Read more information on sharing protocols at https://plos.org/protocols?utm_medium=editorial-email&utm_source=authorletters&utm_campaign=protocols.

As the corresponding author, your ORCID iD is verified in the submission system and will appear in the published article. PLOS supports the use of ORCID, and we encourage all coauthors to register for an ORCID iD and use it as well. Please encourage your coauthors to verify their ORCID iD within the submission system before final acceptance, as unverified ORCID iDs will not appear in the published article. *Only*  the individual author can complete the verification step; PLOS staff *cannot*  verify ORCID iDs on behalf of authors.

We look forward to receiving your revised manuscript.

Kind regards,

Tomasz W. Kaminski

Academic Editor

PLOS One

Journal Requirements:

When submitting your revision, we need you to address these additional requirements.

1. Please ensure that your manuscript meets PLOS ONE's style requirements, including those for file naming. The PLOS ONE style templates can be found at

https://journals.plos.org/plosone/s/file?id=wjVg/PLOSOne_formatting_sample_main_body.pdf and

https://journals.plos.org/plosone/s/file?id=ba62/PLOSOne_formatting_sample_title_authors_affiliations.pdf

2. We note that the grant information you provided in the ‘Funding Information’ and ‘Financial Disclosure’ sections do not match.

When you resubmit, please ensure that you provide the correct grant numbers for the awards you received for your study in the ‘Funding Information’ section.

3. If the reviewer comments include a recommendation to cite specific previously published works, please review and evaluate these publications to determine whether they are relevant and should be cited. There is no requirement to cite these works unless the editor has indicated otherwise.

Additional Editor Comments:

Dear Authors,

Thank you for submitting this interesting feasibility study evaluating intratracheal microspray delivery of iohexol for pulmonary micro-CT imaging.

The approach is promising, and the manuscript is generally well organized; however, several important issues must be addressed before the work can be considered for publication. In particular, the main imaging findings require quantitative support, and the exploratory nature of the small study should be more clearly emphasized. The limitations related to pulmonary safety, potential bias, and the use of male SCID mice should also be discussed more explicitly. For these reasons, the manuscript requires major revision.

Sincerely,

Tomasz W. Kaminski

[Note: HTML markup is below. Please do not edit.]

Reviewers' comments:

Reviewer's Responses to Questions

**Comments to the Author**

1. Is the manuscript technically sound, and do the data support the conclusions?

Reviewer #1: Yes

Reviewer #2: Yes

**********

2. Has the statistical analysis been performed appropriately and rigorously?

Reviewer #1: No

Reviewer #2: Yes

**********

3. Have the authors made all data underlying the findings in their manuscript fully available?

The PLOS Data policy

Reviewer #1: Yes

Reviewer #2: Yes

**********

4. Is the manuscript presented in an intelligible fashion and written in standard English?

Reviewer #1: Yes

Reviewer #2: Yes

**********

Reviewer #1: The manuscript presents a feasibility study evaluating intratracheal microspray delivery of iohexol for prolonged pulmonary enhancement in preclinical micro-CT imaging. The concept of redirecting a clinically available iodinated contrast agent to the airway rather than the intravenous route is interesting and has potential value for longitudinal pulmonary imaging studies. The manuscript is generally well organized, and the authors appropriately acknowledge several limitations of this pilot study.

However, several issues should be addressed before the manuscript is suitable for publication.

Major Comments

1. The primary imaging outcome is evaluated only qualitatively.

The main conclusion of the study is that intratracheal microspray delivery provides sustained pulmonary enhancement compared with intravenous administration. However, this conclusion is based almost entirely on representative images. Quantitative image analysis (e.g., ROI-based Hounsfield unit measurements, time-density curves, or signal intensity comparisons) is not provided. Without objective measurements, it is difficult to assess the magnitude and reproducibility of the reported improvement. If quantitative analysis cannot be added, this limitation should be more explicitly emphasized, and the conclusions should be appropriately tempered.

2. The sample size is small and the study should clearly be presented as exploratory.

Each experimental group contains only 4–5 animals, and no a priori power calculation was performed. While this is understandable for a pilot feasibility study, the limited statistical power should be discussed more explicitly, and the findings should be described as hypothesis-generating rather than definitive.

3. The safety assessment is limited to acute systemic toxicity.

The serum biochemical analyses and histopathology demonstrate no obvious acute toxicity, which is reassuring. However, local pulmonary inflammatory responses and pulmonary function were not evaluated. Although these additional experiments may be beyond the scope of the current study, this limitation should be more clearly acknowledged in the Discussion.

Minor Comments

1. The manuscript would benefit from a more detailed discussion comparing this approach with previously reported long-circulating or nanoparticle-based CT contrast agents, highlighting the relative advantages and limitations of using a clinically available iodinated contrast agent.

2. The lack of randomization and blinded image assessment should be acknowledged as potential sources of bias when interpreting the qualitative imaging results.

3. Because only SCID mice were used, the authors should further emphasize that immune-mediated responses and hypersensitivity reactions cannot be evaluated in this model.

Overall Assessment

This study presents an interesting technical approach with potential applications in preclinical thoracic imaging. However, the principal conclusions rely primarily on qualitative observations, and the manuscript should more clearly acknowledge the exploratory nature of the work. With appropriate revision of the discussion and a more cautious interpretation of the findings, the manuscript would provide a useful contribution to the field.

Reviewer #2: Summary

This feasibility study in male SCID mice compares intratracheal microspray instillation versus conventional IV tail-vein injection of iohexol for preclinical thoracic micro-CT, addressing the well-known limitation of the brief pulmonary enhancement window with IV contrast. The authors show that intratracheal delivery produces sustained, comparatively uniform lung-parenchymal opacification over 120 minutes, versus transient vascular signal and rapid renal clearance with IV delivery. Acute systemic safety (serum biochemistry at 5 and 29 h, histopathology of five major organs) was comparable between routes, with only a small, non-route-specific increase in creatinine. The study is explicitly framed as hypothesis-generating pilot data.

Strengths

- Clear rationale, well-matched methods, and appropriately scoped conclusions throughout — the authors do not overstate what a small qualitative pilot can support.

- Statistical approach is well-matched to the design: two-way ANOVA with group and time as independent fixed factors is correct given that each animal contributes serum data at only one terminal time point (cardiac blood draw at euthanasia), and the exploratory nature of the biochemistry analysis is clearly disclaimed, including the lack of correction across the four analytes.

- The Limitations are unusually thorough and specific, going well beyond generic caveats. This was greatly appreciated.

- Strain choice (SCID) is justified by the parent xenograft imaging programme rather than arbitrary, and its interpretive consequences (no hypersensitivity assessment, male-only cohort) are flagged early and revisited in the Limitations.

Minor / optional suggestions

- The rationale for using only male animals is stated as a limitation but not explained in the Methods (e.g., colony availability, programme convention). A one-line clarification would tie this off more completely.

- Given the small per-cell sample size (n = 4-5 across 3 groups x 2 time points), consider briefly noting whether variance homogeneity was checked, since Tukey HSD and the pooled variance estimate assume roughly equal variance across cells.

- Since creatinine was the only significant finding among four analytes without cross-analyte correction, consider stating explicitly in the Results or Discussion (not only the Statistics methods) that this finding should be interpreted as exploratory.

Recommendation

- Minor revision. The work is well-conducted and honestly reported for its stated scope; the above points are polish rather than substantive concerns.

**********

what does this mean?). If published, this will include your full peer review and any attached files.

If you choose “no”, your identity will remain anonymous but your review may still be made public.

**Do you want your identity to be public for this peer review?**  For information about this choice, including consent withdrawal, please see our Privacy Policy

Reviewer #1: No

Reviewer #2: No

**********

[NOTE: If reviewer comments were submitted as an attachment file, they will be attached to this email and accessible via the submission site. Please log into your account, locate the manuscript record, and check for the action link "View Attachments". If this link does not appear, there are no attachment files.]

To ensure your figures meet our technical requirements, please review our figure guidelines: https://journals.plos.org/plosone/s/figures

You may also use PLOS’s free figure tool, NAAS, to help you prepare publication quality figures: https://journals.plos.org/plosone/s/figures#loc-tools-for-figure-preparation.

NAAS will assess whether your figures meet our technical requirements by comparing each figure against our figure specifications.

# Author comment: Author response to Decision Letter 1

**DOI:** 10.1371/journal.pone.0361103.r002

20 Sep 2026

Reviewer #1:

Major Comments

1. The primary imaging outcome is evaluated only qualitatively. Quantitative image analysis (e.g., ROI-based Hounsfield unit measurements, time-density curves, or signal intensity comparisons) is not provided.

Response: We agree that quantitative measurements strengthen the conclusion and have added them. Regions of interest were placed on the bladder and lung field in the whole-body projections (Fig 2B, 2C) and on the central thoracic region in the coronal and axial sections (Fig 3C, 3D) at every time point, for four animals per group. Because no density calibration phantom was scanned, signal is expressed as normalized relative signal in arbitrary units (a.u.), with each animal’s pre-contrast scan as its own baseline; this is the signal-intensity comparison suggested by the Reviewer. The quantification confirms the images: intratracheal delivery gave a coronal thoracic signal of 0.77 a.u. at 5 min and 0.72 a.u. at 30 min, against 0.09 to 0.19 a.u. after IV injection. All individual values are provided in S1 Data.

“Regions of interest (ROIs) were placed on the urinary bladder and the lung field in the whole-body projections, and on the central thoracic region in the coronal and axial sections, at every time point. The mean gray-level intensity of each ROI was extracted in Python 3.” (Materials and methods, Image assessment, page 8, lines 150-152)

“In the coronal view (Fig 3C), normalized signal in the intratracheal group was 0.77 a.u. (range 0.70 to 0.85) at 5 min, 0.70 a.u. (0.63 to 0.73) at 15 min and 0.72 a.u. (0.64 to 0.77) at 30 min, against 0.09 a.u. (0.08 to 0.11), 0.19 a.u. (0.15 to 0.26) and 0.18 a.u. (0.13 to 0.25) for the IV group.” (Results, Pulmonary contrast enhancement, page 11, lines 216-219)

2. The sample size is small and the study should clearly be presented as exploratory. The limited statistical power should be discussed more explicitly, and the findings should be described as hypothesis-generating rather than definitive.

Response: We agree that the scope of the study should be clear. The study is presented throughout as a feasibility comparison, and the findings as the basis for a confirmatory quantitative study. The Discussion states that group sizes were not set by a power calculation and how the confirmatory study should be powered, and the Conclusions name that study as the next step. The direction and location of the effect were consistent in every animal (Figs 2 and 3; S1 Data), which is what a feasibility study is designed to establish.

“Group sizes were small (n = 4 per group for imaging, n = 5 for serum) and were not set by a power calculation; the study was designed to establish feasibility and the direction of effect, which it does, and the confirmatory study should be powered from calibrated measurements.” (Discussion, pages 16 to 17, lines 341-344)

“A powered, quantitative, blinded study in immunocompetent animals of both sexes, acquired with a density calibration phantom, is the logical next step to quantify the attenuation difference and to define the duration of parenchymal persistence beyond 30 min.” (Conclusions, page 17, lines 359-361)

3. The safety assessment is limited to acute systemic toxicity. Local pulmonary inflammatory responses and pulmonary function were not evaluated; this limitation should be more clearly acknowledged in the Discussion.

Response: The Discussion now states what the safety assessment covers, names the pulmonary endpoints not evaluated, and identifies them as endpoints of the confirmatory study.

“The safety data cover acute systemic tolerance; local pulmonary inflammatory responses, lung mechanics, mucociliary clearance and repeat-dosing effects were not assessed, and bronchoalveolar lavage and lung-function endpoints are planned for the confirmatory study.” (Discussion, page 16, lines 329-332)

Minor Comments

1. The manuscript would benefit from a more detailed discussion comparing this approach with previously reported long-circulating or nanoparticle-based CT contrast agents, highlighting the relative advantages and limitations of using a clinically available iodinated contrast agent.

Response: We appreciate this suggestion and have expanded the comparison in the Discussion. Blood-pool agents prolong enhancement of the pulmonary vasculature, whereas intratracheal delivery fills the airspace, so the two strategies address different imaging questions and are complementary [1]. The advantages of a clinically available agent (established dose range and safety profile, no formulation development) and its limitation (clearance from the airspace sets the imaging window) are now stated.

“Its advantages are that the agent, dose range and safety profile are already established clinically and no formulation development is required; its limitation is that clearance from the airspace sets the imaging window, which here comfortably exceeded the 30-min tomographic series.” (Discussion, pages 14 to 15, lines 297-300)

2. The lack of randomization and blinded image assessment should be acknowledged as potential sources of bias when interpreting the qualitative imaging results.

Response: Thank you for this concern. We have added the procedure to the Methods and Discussion. Animals were assigned to route in the order they became available, and each animal was scanned before contrast in the same session as its own control. The CT examinations were acquired by a facility technician who did not know the group allocation. The images were then quantified by two readers, a pulmonologist blinded to group and time point and a micro-CT-trained investigator who was not blinded; their results were concordant and were averaged for analysis. Randomized allocation and fully blinded scoring are specified for the confirmatory study.

“Image acquisition was performed by a facility technician who was unaware of the group allocation.” (Materials and methods, Micro-computed tomography, page 7, lines 147-148)

“Quantification was performed independently by two readers, a pulmonologist blinded to group and time point and a micro-CT-trained investigator who was not blinded; the two sets of measurements were concordant and were averaged for analysis.” (Materials and methods, Image assessment, page 8, lines 156-158)

3. Because only SCID mice were used, the authors should further emphasize that immune-mediated responses and hypersensitivity reactions cannot be evaluated in this model.

Response: This point is now stated in the Discussion, and the confirmatory study design specifies immunocompetent animals of both sexes.

“SCID mice lack adaptive immunity, so hypersensitivity reactions to iodinated contrast cannot be evaluated in this strain, and only male mice were used.” (Discussion, page 17, lines 344-346)

Reviewer #2:

We are grateful for the positive evaluation and have addressed each suggestion below.

1. The rationale for using only male animals is stated as a limitation but not explained in the Methods. A one-line clarification would tie this off more completely.

Response: Thank you for this suggestion. A one-line explanation has been added to the Methods.

“Male mice were used to reduce experimental variability arising from the estrous cycle and hormonal fluctuation in female mice.” (Materials and methods, Animals, group allocation, and contrast delivery, page 6, lines 127-128)

2. Given the small per-cell sample size, consider briefly noting whether variance homogeneity was checked, since Tukey HSD and the pooled variance estimate assume roughly equal variance across cells.

Response: Variance homogeneity was assessed by inspecting the per-cell standard deviations, which are given in S1 Data, and this is now stated in the Statistical analysis section. The creatinine effect is large relative to the within-cell spread (treated means exceed the control mean by 0.06 to 0.10 mg/dL, against standard deviations of 0.01 to 0.06 mg/dL) and is present at both time points, and the between-route comparison, which involves the two cells with similar spread, was not significant. The conclusions do not depend on the equal-variance assumption.

“Variance homogeneity was assessed by inspection of the per-cell standard deviations (S1 Data).” (Materials and methods, Statistical analysis, page 9, lines 176-177)

3. Since creatinine was the only significant finding among four analytes without cross-analyte correction, consider stating explicitly in the Results or Discussion that this finding should be interpreted as exploratory.

Response: We agree. The statement has been added to the Results, and the Discussion now explains the finding. Iohexol is eliminated unchanged by glomerular filtration and is itself used as a marker of glomerular filtration rate [2]. Iohexol deposited in the airspace is absorbed into the circulation and excreted by the same path, which explains why both routes produced the same small increase. The increase (0.06 to 0.10 mg/dL) is well below the 0.3 mg/dL rise that defines post-contrast acute kidney injury [3,4], and BUN and renal histology were unchanged, so it is interpreted as a transient functional response to renal excretion of the agent rather than injury.

“This small, route-independent increase is consistent with renal excretion of absorbed iohexol; BUN and renal histology were unchanged (Fig 5), and as the only significant result among four analytes tested without cross-analyte correction it is regarded as an exploratory observation.” (Results, Systemic safety and histology, page 12, lines 244-247)

“Iohexol is eliminated unchanged by glomerular filtration and is itself used as an exogenous marker of glomerular filtration rate [19]; iohexol deposited in the airspace is absorbed into the circulation and excreted by the same path, consistent with the early bladder signal after instillation (Fig 2B), which explains why the two routes behaved alike.” (Discussion, pages 15 to 16, lines 320-324)

References

1. Hallouard F, Anton N, Choquet P, Constantinesco A, Vandamme T. Iodinated blood pool contrast media for preclinical X-ray imaging applications: a review. Biomaterials. 2010;31: 6249-6268.

2. Dong Q, Chen Z, Boland J, Dokos C, Hagos Y, Kühne A, et al. Validating low-dose iohexol as a marker for glomerular filtration rate by in vitro and in vivo studies. Clin Transl Sci. 2025;18: e70141.

3. Davenport MS, Perazella MA, Yee J, Dillman JR, Fine D, McDonald RJ, et al. Use of intravenous iodinated contrast media in patients with kidney disease: consensus statements from the American College of Radiology and the National Kidney Foundation. Radiology. 2020;294: 660-668.

4. van der Molen AJ, Reimer P, Dekkers IA, Bongartz G, Bellin MF, Bertolotto M, et al. Post-contrast acute kidney injury, Part 1: definition, clinical features, incidence, role of contrast medium and risk factors. Eur Radiol. 2018;28: 2845-2855.

Attachment Submitted filename: Response_to_Reviewers.docx

# Peer review: Decision Letter 1

**DOI:** 10.1371/journal.pone.0361103.r003

**Authors:** Tomasz Kaminski

7 Oct 2026

Intratracheal microspray delivery of iohexol for sustained lung-parenchymal enhancement in preclinical micro-computed tomography: a feasibility and acute-safety study in mice

PONE-D-26-30016R1

Dear Dr. Tseng,

We’re pleased to inform you that your manuscript has been judged scientifically suitable for publication and will be formally accepted for publication once it meets all outstanding technical requirements.

Within one week, you’ll receive an e-mail detailing the required amendments. When these have been addressed, you’ll receive a formal acceptance letter and your manuscript will be scheduled for publication.

An invoice will be generated when your article is formally accepted. Please note, if your institution has a publishing partnership with PLOS and your article meets the relevant criteria, all or part of your publication costs will be covered. Please make sure your user information is up-to-date by logging into Editorial Manager at Editorial Manager® and clicking the ‘Update My Information' link at the top of the page. For questions related to billing, please contact billing support.

If your institution or institutions have a press office, please notify them about your upcoming paper to help maximize its impact. If they’ll be preparing press materials, please inform our press team as soon as possible -- no later than 48 hours after receiving the formal acceptance. Your manuscript will remain under strict press embargo until 2 pm Eastern Time on the date of publication. For more information, please contact onepress@plos.org.

Kind regards,

Tomasz W. Kaminski

Academic Editor

PLOS One

Reviewers' comments:

Reviewer's Responses to Questions

**Comments to the Author**

Reviewer #1: All comments have been addressed

Reviewer #2: All comments have been addressed

**********

2. Is the manuscript technically sound, and do the data support the conclusions?

Reviewer #1: Yes

Reviewer #2: Yes

**********

3. Has the statistical analysis been performed appropriately and rigorously?

Reviewer #1: Yes

Reviewer #2: Yes

**********

4. Have the authors made all data underlying the findings in their manuscript fully available?

The PLOS Data policy

Reviewer #1: (No Response)

Reviewer #2: Yes

**********

5. Is the manuscript presented in an intelligible fashion and written in standard English?

Reviewer #1: Yes

Reviewer #2: Yes

**********

Reviewer #1: The authors have adequately addressed the comments raised in the previous review. The revised manuscript is clearer, and the limitations of this feasibility study are now appropriately acknowledged. I have no further concerns and consider the manuscript suitable for publication.

Reviewer #2: Dear Authors,

Thank you for the careful and constructive revision. Your responses addressed each of my comments, and the additions to the Methods, Statistical analysis, Results, and Discussion are clear and well supported. In particular, framing the creatinine finding as exploratory, alongside the explanation based on renal excretion of absorbed iohexol, gives readers a sensible way to interpret it.

I have no further requests. I wish you the best with the publication.

Kind regards,

Reviewer #2

**********

what does this mean?). If published, this will include your full peer review and any attached files.

If you choose “no”, your identity will remain anonymous but your review may still be made public.

**Do you want your identity to be public for this peer review?**  For information about this choice, including consent withdrawal, please see our Privacy Policy

Reviewer #1: No

Reviewer #2: No

**********

# Editor report: Acceptance letter

**DOI:** 10.1371/journal.pone.0361103.r004

**Authors:** Tomasz Kaminski

PONE-D-26-30016R1

PLOS One

Dear Dr. Tseng,

I'm pleased to inform you that your manuscript has been deemed suitable for publication in PLOS One. Congratulations! Your manuscript is now being handed over to our production team.

At this stage, our production department will prepare your paper for publication. This includes ensuring the following:

* All references, tables, and figures are properly cited

* All relevant supporting information is included in the manuscript submission,

* There are no issues that prevent the paper from being properly typeset

You will receive further instructions from the production team, including instructions on how to review your proof when it is ready. Please keep in mind that we are working through a large volume of accepted articles, so please give us a few days to review your paper and let you know the next and final steps.

Lastly, if your institution or institutions have a press office, please let them know about your upcoming paper now to help maximize its impact. If they'll be preparing press materials, please inform our press team within the next 48 hours. Your manuscript will remain under strict press embargo until 2 pm Eastern Time on the date of publication. For more information, please contact onepress@plos.org.

You will receive an invoice from PLOS for your publication fee after your manuscript has reached the completed accept phase. If you receive an email requesting payment before acceptance or for any other service, this may be a phishing scheme. Learn how to identify phishing emails and protect your accounts at https://explore.plos.org/phishing.

If we can help with anything else, please email us at customercare@plos.org.

Thank you for submitting your work to PLOS One and supporting open access.

Kind regards,

PLOS One Editorial Office Staff

on behalf of

Dr. Tomasz W. Kaminski

Academic Editor

PLOS One
